# Development of a new method for identification and quantification in cerebrospinal fluid of malignant cells from breast carcinoma leptomeningeal metastasis

**DOI:** 10.1186/1472-6890-12-21

**Published:** 2012-11-12

**Authors:** Emilie Le Rhun, Frédéric Massin, Qian Tu, Jacques Bonneterre, Marcelo De Carvalho Bittencourt, Gilbert C Faure

**Affiliations:** 1 Neurology, Breast Unit, Deparment of Medical Oncology, Oscar Lambret Center, Lille, France and Neuro-oncology, University Hospital, Lille, France; 2 CHU Nancy, Nancytomique, Laboratoire d’Immunologie, Pôle Laboratoires, Vandoeuvre lès Nancy, Nancy, France; 3 CHU Nancy, Nancytomique, Laboratoire d’Immunologie, Pôle Laboratoires, Vandoeuvre lès Nancy, Nancy, France; 4 Breast Unit, Department of Medical Oncology, Centre Oscar Lambret, Lille, France and University of Lille Nord de France, Lille, France; 5 CHU Nancy, Nancytomique, Laboratoire d’Immunologie, Pôle Laboratoires, Vandoeuvre lès Nancy, France and Université Henri Poincaré, Faculté de Médecine, Vandoeuvre lès Nancy, EA4369 RHEM, Nancy, France; 6 CHU Nancy, Nancytomique, Laboratoire d’Immunologie, Pôle Laboratoires, Vandoeuvre lès Nancy, France and Université Henri Poincaré, Faculté de Médecine, Vandoeuvre lès Nancy, EA4369 RHEM, Nancy, France

**Keywords:** Leptomeningeal metastasis, Neoplastic meningitis, Meningeal carcinomatosis, CellSearch® technology, Immunomagnetic enrichment, CSF cytology

## Abstract

**Background:**

The diagnosis of leptomeningeal metastasis (LM) in patients with solid tumors remains difficult. The usual diagnostic methods of cytomorphological assessment of cerebro-spinal fluid (CSF) and gadolinium enhanced MRI of the entire neuraxis lack both specificity and sensitivity. The Veridex CellSearch® technology has been designed for the detection of circulating tumor cells (CTC) in blood from cancer patients and validated for the follow-up and prognosis of breast, prostate, colorectal, and lung cancer. Our aim was to adapt this technology for the detection and the enumeration of tumor cells in the CSF of breast cancer patients presenting with LM.

**Methods:**

On the occasion of a randomized phase III study evaluating the role of the intrathecal treatment in LM from breast cancer (DEPOSEIN, EudraCT N°: 2010-023134-23), the CellSearch® technology was adapted to direct enrichment, enumeration and visualization of tumor cells in 5 mL CSF samples, collected on CellSave® Preservative Tubes and analyzed within 3 days after CSF sampling.

**Results:**

Sixteen CSF of 8 patients with primary breast cancer presenting with LM were studied. EpCAM+/cytokeratin + cells with typical morphology could be observed and enumerated sequentially with reproducible results in low or elevated numbers in 8 patients.

**Conclusion:**

This methodology, established on a limited volume of sample and allowing delayed processing, could prove of great interest in the diagnosis and follow-up of cancer patients with LM, especially to appreciate the efficacy of chemotherapy.

## Background

Solid tumors, mostly breast cancer, lung cancer and melanoma, can result in leptomeningeal metastasis (LM) in 5 to 19% of patients [1]. The incidence of central nervous system (CNS) metastasis and LM may increase in the coming years because of a prolonged control of extra-cerebral disease and because of the use of antineoplastic agents with a poor diffusion into the CNS [2].

The median survival of untreated patients with LM is 4–6 weeks. Breast cancers LM have the best prognosis, and median overall survival may reach 3 to 5 months with a combined treatment in recent studies [3-8]. The aim of treatment is to improve or stabilize neurological functions, maintain quality of life and prolong survival [1].

Prognostic factors have been identified: age, performance status, neurological status, LM characteristics, cerebrospinal fluid (CSF) block, LM related encephalopathy, extension of systemic disease and its treatment options, interval between diagnosis of primary tumour and LM and type of primary tumour [1,9]. These factors remain heterogeneous among studies and are not very well validated. LM should be diagnosed in the early stages of the disease to prevent the progression of disabling neurological deficits. The diagnosis is assessed by CSF cytomorphological analysis or by concomitant typical CNS involvement symptoms and gadolinium enhanced magnetic resonance imaging (MRI) signs. CNS signs and symptoms, indicative of LM in more than 90% of patients, may be pleomorphic and are often subtle and difficult to distinguish from other cancer or antineoplastic treatment complications [10]. The specificity of gadolinium-enhanced MRI signs is up to 100% in solid tumors, balanced by a risk of false negative as high as 65% and false positive approaching 10% [11]. Evidence of malignant cells in the CSF is diagnostic of LM. However, in patients ultimately positive for CSF cytology, up to 45% are cytologically negative on initial examination. The sensitivity reaches 80% with a second CSF analysis, but little benefit is obtained from more than two repeated lumbar punctures [1]. Insufficient CSF sampling, collecting CSF at distant site of symptoms and delayed processing have been reported to be sources of error [10-13]. Various biomarkers, such as tumour antigens, molecules involved in extravasation, migration or angiogenesis as well as chemokines [14-18] are under evaluation for their performance in detecting LM. Flow cytometry has been proposed as well as cytomics approaches, especially for CNS haematological involvement [17,19].

CSF cytomorphology thus remains the gold standard for LM detection, but readout is only qualitative and not quantitative. Besides microscopic enumeration and morphological examination, cellular biomarkers appear promising.

The Veridex CellSearch® technology has been designed for the detection of circulating tumour cells (CTC) in blood from cancer patients and validated for the follow-up and prognosis of breast, prostate, colorectal, and lung cancer [20-22]. We adapted the technique and applied it to detect malignant cells in the CSF of 8 patients with breast cancer LM.

## Methods

All the patients were included into a randomized phase 3 study evaluating the role of the intrathecal treatment in LM from breast cancer (DEPOSEIN, EudraCT N°: 2010-023134-23) after approbation of the appropriate regional ethic committee (“Comité de Protection des Personnes - CPP Nord Ouest III) on May 3rd 2011 and in compliance with the Helsinki Declaration. All patients had given their written informed consent for the translational study. DEPOSEIN is a multicentric randomized phase III study designed to assess the role of intra-CSF liposomal cytarabine in the treatment of LM. Patients are randomized in 2 arms: liposomal cytarabine intra-CSF and concomitant systemic treatment versus systemic treatment alone. LM diagnosis was established in 8 patients with primary breast cancer, according to usual diagnostic criteria. The CellSearch® technology defines CTCs after immunomagnetic enrichment of cells expressing EpCAM, using nuclear staining with 4’, 6-diamidino-2- phenylindole (DAPI), and immunofluorescence detection of cytokeratin and CD45 [22]. Five mL of CSF samples were collected on CellSave® Preservative Tubes (Veridex, Raritan, NJ) and analyzed within 3 days after CSF sampling using the standard CellSearch® protocol and the CTC Epithelial Cell Kit (Veridex, Raritan, NJ). The methodology is designed to initially eliminate, in centrifuged blood samples, the plasma above the buffy coat and erythrocytes by optically detecting the level of the latter. In order to avoid undue alarms, after deposition of 5 mL of CSF in the conic tube of the “circulating tumor cell kit”; the outside of the tube was simply darkened with a black felt-tip up to the fluid level to mimick the erythrocytes level. Five mL of dilution buffer were then added and the mixture homogeneized and centrifuged. The “lured-tube” was then placed into the preparation station and submitted to automate preparation as for blood samples.

Briefly, the latter resulted in an enriched sample where cells were optically aligned along the upper panel of a glass chamber, maintained by strong magnets, allowing for automated fluorescence microscopy and cell images digitalization, a cell being defined as both nuclear DAPI and cell-surface staining. This allowed the observation of EpCAM+/cytokeratin + cells with a DAPI-stained nucleus and no CD45 staining, magnetically maintained in a single plane for electronic image analysis and digitalization. The automated fluorescence microscope proposes galleries of cells, which were validated upon visual image review. DAPI+/CD45+/EpCAM-/cytokeratin- cells were counted as leukocytes.

In parallel, cytocentrifuge smears of each CSF sample were prepared within one hour after CSF sampling and stained (May Grunwald Giemsa) for visual cytomorphological examination of tumor cells, and standard CSF biochemical composition was analyzed.

## Results

The characteristics of the patients at the time of LM diagnosis are described in Table 1. Five patients had concomitant parenchymal brain metastasis. At LM diagnosis, 6 patients presented with symptoms and signs, mostly including cauda equina syndrome and dizziness. Proteinorachia varied from 0.24 to 7.08 g/L. CSF samples volumes varied between 5 and 10 mL for usual cytomorphological analysis, tumor cells were detected but not enumerated in all the patients and MRI showed evidence of LM signs in 8 patients.

**Table 1:** Patients’ characteristics at LM diagnosis

**Number of inclusion**	**Age at BC diagnosis**	**BC characteristics**	**Age at LM diagnosis**	**Clinical symptoms**	**Biochemical CSF analysis**	**Cytological CSF analysis**	**Neuraxis MRI**
03	56	Undifferenciated carcinoma, HPG 3, ER-, PR -, HER2-	60	Cognitive disorders	Proteinorachia: 0.24 g/l	Presence of malignant cells	LM involvement
					Glycorachia: 2.7		Brain metastases
					Chlorurorachia: 118		
05	61	ILC, HPG 2, ER+, PR+, HER2-	61	Dizziness	Proteinorachia: 2.19 g/l	Presence of malignant cells	LM involvement
					Glycorachia: 1.7		No brain metastases
					Chlorurorachia: 124		
06	53	Adenocarcinoma, HPG 2, ER+, PR+, HER2 -	74	None	Proteinorachia: 0.48 g/l	Presence of malignant cells	LM involvement
					Glycorachia: 3.5		No brain metastases
					Chlorurorachia: 123		
09	42	Adenocarcinoma, HPG 2, ER+, PR +, HER2 -	60	Dizziness, deafness	Proteinorachia: 1.49 g/l	Presence of malignant cells	LM involvement
					Glycorachia: 2.7		No brain metastases
					Chlorurorachia: 116		
10	47	IDC, HPG 2, ER+, PR+, HER2-	59	Visual disorders	Proteinorachia: 0.51 g/l	Presence of malignant cells	LM involvement
					Glycorachia: 4.0		Brain metastases
					Chlorurorachia: 120		
11	41	ICL, HPG 3, ER+, PR -, HER2 +	45	Cauda equine syndrome	Proteinorachia: 7.08 g/l	Presence of malignant cells	LM involvement
					Glycorachia: 2		Brain metastases
					Chlorurorachia: 113		
12	44	IDC, HPG 2, ER+, PR+, HER2 +	57	None	Proteinorachia: 0.28 g/l	Presence of malignant cells	LM involvement
					Glycorachia: 4.9		Brain metastases
					Chlorurorachia: 120		
13	45	Undifferenciated carcinoma, HPG 3, ER+, PR+, HER2 -	50	Cauda equina syndrome	Proteinorachia: 5.80 g/l	Presence of malignant cells	LM involvement
					Glycorachia: 3.6		Brain metastases
					Chlorurorachia: 111		

*LM* Leptomeningeal Metastases, *CSF* Cerebro Spinal Fluid, *TC* Tumor Cells, *ILC* Invasive Lobular Carcinoma, *IDC* Invasive Ductal Carcinoma, *HPG* histoprognostic grade, *ER* Estrogen Receptors, *PR* Progesterone Receptors, *HER2* Human Epidermal Growth Factor Receptor-2, *TTF1* Thyroid Transcription Factor 1, *EGFR* Epidermal Growth Factor Receptor, *CNS* Central Nervous System, *MRI* Magnetic Resonance Imaging, IT IntraThecal.

**Figure 1:**
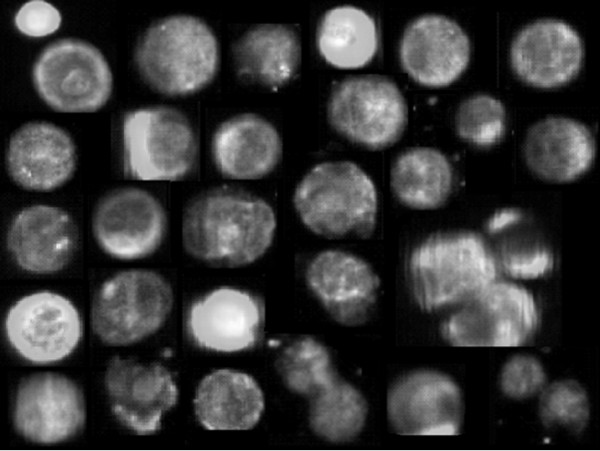
Collection of validated images of tumor cells (EpCAM+/cytokeratin + cells with a DAPI-stained nucleus and no CD45 staining) detected in the CSF from a patient with LM secondary to breast cancer.

**Figure 2:**
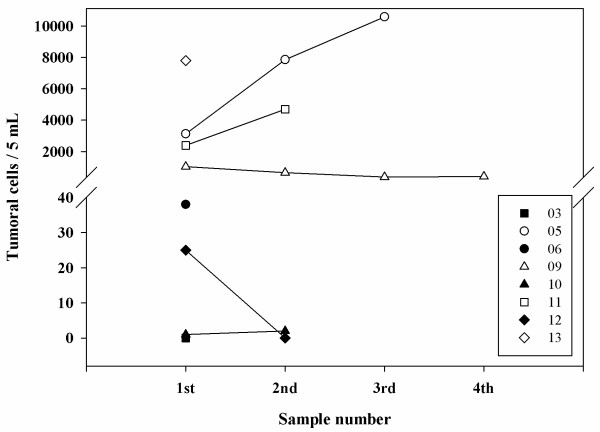
Individual numbers of tumor cells in CSF detected using the CellSearch® technology from the 8 patients included in the study (identified by their number of inclusion at the right box).

The first part of the translational study was to adapt the CellSearch® technology for the detection and enumeration of tumor cells in the CSF of breast cancer LM. Sixteen CSF samples were available for the evaluation of the innovative method: These samples are described in Table 2. All CSF samples were obtained at lumbar site and analysed at LM diagnosis or at different times during LM treatment. Malignant cells were observed at cytomorphological analysis in 12 samples, and in 14 samples with the CellSearch® technology, with tumor cells numbers ranging between 1 and 10500 /5 mL. Figure 1 shows representative epithelial tumour cells detected in CSF from one breast cancer patient by CellSearch® technology. These cells have similar aspects to epithelial tumour cells described in blood as CTC from breast cancer patients [17]. Figure 2 summarizes quantitative results of tumor cells in CSF by CellSearch® technology.

**Table 2:** CSF characteristics at the time of the CellSearch® technology analysis

**Inclusion number, date of CSF sampling**	**Treatment received for LM at the time of CSF sampling for CVT**	**Biochemical CSF analysis**	**Cytomorphological CSF analysis**	**Enumeration of CSFTC with the CellSearch® Veridex technology**
03 - 01	IV paclitaxel for 2 months then	Proteinorachia: 0.22 g/L	No malignant cell (10 mL)	0 CSFTC per mL
	carboplatin	Glycorachia: 3.5 mmol/L		
	No IT chemotherapy	Chlorurorachia: 121 mol/L		
05 - 01	IV paclitaxel since LM diagnosis	Proteinorachia: 1.13 g/L	Presence of malignant cells (10 mL)	626 CSFTC per mL
	No IT chemotherapy	Glycorachia: 2.4 mmol/L		
		Chlorurorachia: 121 mol/L		
05 - 02	IV paclitaxel since LM diagnosis	Proteinorachia: 1.42 g/L	Presence of malignant cells (10 mL)	1600 CSFTC per mL
	No IT chemotherapy	Glycorachia: 1.8 mmol/L		
		Chlorurorachia: 117 mol/L		
05 - 03	IV paclitaxel since LM diagnosis	Proteinorachia: 2.90 g/L	No malignant cell (10 mL)	2100 CSFTC per mL
	No IT chemotherapy	Glycorachia: 0.3 mmol/L		
		Chlorurorachia: 118 mol/L		
06 - 01	None	Proteinorachia: 0.51 g/L	Presence of malignant cells (3 mL)	7 CSFTC per mL
		Glycorachia: 3.7 mmol/L		
		Chlorurorachia: 126 mol/L		
09 - 01	None	Proteinorachia: 1.90 g/L	Presence of malignant cells (6 mL)	208 CSFTC per mL
		Glycorachia: 3.3 mmol/L		
		Chlorurorachia: 110 mol/L		
09 - 02	Oral vinorelbine	Proteinorachia: 1.64 g/L	Not interpretable (10 mL)	130 CSFTC per mL
	IT liposomal cytarabine (X1)	Glycorachia: 4.9 mmol/L		
		Chlorurorachia: 108 mol/L		
09 -03	Oral navelbine	Proteinorachia: NA	Presence of malignant cells (10 mL)	75 CSFTC per mL
	IT liposomal cytarabine (X2)	Glycorachia: NA		
		Chlorurorachia: NA		
09 -04	Oral navelbine	Proteinorachia: 1.88 g/L	Presence of malignant cells (10 mL)	82 CSFTC per mL
	IT liposomal cytarabine (X3)	Glycorachia: 4.5 mmol/L		
		Chlorurorachia: 116 mol/L		
10 - 01	None	Proteinorachia: 0.80 g/L	Presence of malignant cells (5 mL)	0.2 CTC per mL
		Glycorachia: 3.4 mmol/L		
		Chlorurorachia: 121 mol/L		
10 - 02	FEC 50	Proteinorachia: 0.90 g/L	Presence of malignant cells (6 mL)	0.4 CSFTC per mL
	IT liposomal cytarabine (X1)	Glycorachia: 3.2 mmol/L		
		Chlorurorachia: 120 mol/L		
11 - 01	None	Proteinorachia: 7.08 g/L	Presence of malignant cells (6 mL)	478 CSFTC per mL
		Glycorachia: 2 mmol/L		
		Chlorurorachia: 113 mol/L		
11 - 02	Capecitabine	Proteinorachia: 9.04 g/L	Presence of malignant cells (8 mL)	940 CSFTC per mL
	No IT chemotherapy	Glycorachia: 3.2 mmol/L		
		Chlorurorachia: 100 mol/L		
12 - 01	None	Proteinorachia: 0.39 g/L	Presence of malignant cells (10 mL)	5 CSFTC per mL
		Glycorachia: 5.4 mmol/L		
		Chlorurorachia: 119 mol/L		
12 - 02	Oral capecitabine + lapatinib	Proteinorachia: 0.21 g/L	No malignant cells (8 mL)	0 CSFTC per mL
	IT liposomal cytarabine (X1)	Glycorachia: 5.4 mmol/L		
		Chlorurorachia: 119 mol/L		
13 - 01	None	Proteinorachia: 8.84 g/L	Presence of malignant cells (7 mL)	1560 CSFTC per mL
		Glycorachia: 2.4 mmol/L		
		Chlorurorachia: 106 mol/L		

*LM* Leptomeningeal Metastasis, *CSF* CerebroSpinal Fluid, *CSFTC* CSF Tumor Cells, *IT* IntraThecal, *IV* IntraVenous, *FEC* Fluorouracil Epirubicin Cyclophosphamide, *CVT* CellSearch® Veridex Technology, *NA* Not Available.

Usual cytological CSF analysis was not interpretable in one sample from patient 09 because of deterioration of the cells, despite an usual procedure of sampling and processing of the CSF, while CellSearch® showed 650 tumor cells in 5 mL. In another patient, the usual cytologic analysis did not detect any tumor cell but CellSearch® technology enumerated more than 2000 cells /mL.

Three patients were studied once with both techniques, with concordant positive and negative results. Two patients were studied twice, one with low levels of cells (1 and 2 cells /5 mL) and the other with elevated numbers (2392 and 4700 cells /5 mL). One patient was analyzed three times with numbers increasing from 3129 to 10500 cells /5 mL. Another patient was studied four times, with numbers of tumor cells decreasing from 1042 cells to 408 cells /5 mL).

## Discussion

In cancer patients, the diagnosis of LM remains a major problem. CNS signs and symptoms orienting towards a diagnosis of LM are often subtle. Diagnosis assessment implies the use of specific techniques, which are not applied in the usual follow-up of solid tumour patients, namely gadolinium enhanced MRI and CSF cytomorphological analyses. Yet, even the latter may be not contributive, as many patients are both radiographically and cytomorphologically negative even in the presence of evocative symptoms.

Different biomarkers have been tested in order to increase the sensitivity of CSF analyses but their use is hampered by a poor specificity/sensitivity, a lack of standardization of CSF sampling and processing, and the absence of agreement on cut-off levels. Because of all these limitations, CSF cytomorphology remains the gold standard for CSF analysis.

The CellSearch® method provides a semi-automated cell analysis, based on the assessment of nuclear and surface markers, which vastly improves the sensitivity, reliability, objectivity, and accuracy of circulating tumor cells detection in the blood compared to cytomorphology [20-22]. In recent studies [23,24], CellSearch® technology, applied by spiking blood with CSF [24], was shown to be of interest for the early detection of CSF malignant cells. We present here a simple modification of this technology allowing for a precise direct quantification of CTC in only 5 mL of CSF. Moreover, the use of CellSave® Preservative tubes for collection authorized a 3-days delay in sample processing, which can be interesting for centralized analyses. The specificity of this technique, besides the use of tumor-associated markers, is enhanced by visual appreciation of the digitalized cells. Indeed, no cells with a tumor-like morphology or phenotype were detected in the CSF of a small series of control patients without LM (data not shown). The presence of an additional free channel for other reagents also makes possible to analyse the cell’s HER2-Neu or EGFR immunofluorescence or to investigate for any other relevant marker allowing the exploration of these neurological metastatic cells.

Furthermore, sequential studies in our short series appear very concordant with either low or elevated levels of cells, which is very encouraging for the evaluation of therapies.

## Conclusions

We report in this pilot study an easy new method for the identification and the quantification of malignant cells in the cerebrospinal fluid. These results, which need to be confirmed in a larger series, suggests that CellSearch® technology could be of interest during cancer patients’ follow-up, for initial characterization of LM and notably to evaluate the efficacy of chemotherapy in its management. According to these first results, the CellSearch® technology will continue to be evaluated during the clinical randomized study DEPOSEIN.

## Competing interests

The authors do not have any potential financial competing interests to declare.

## Authors’ contribution

All authors listed have contributed significantly to the experiment or the writing on the manuscript. ELR conceived the study, participated in its design and coordination, helped to draft the manuscript. FM carried out the cellular immunoassay and acquired data. TQ carried out the cellular immunoassay and acquired data. JB participated in the design of the study and approved the manuscript. MDCB participated in the design of the study and helped to draft the manuscript. GCF designed the study, analysed and interpreted data, helped to draft, revised and approved the manuscript. All authors read and approved the final manuscript.

## Pre-publication history

The pre-publication history for this paper can be accessed here:

http://www.biomedcentral.com/1472-6890/12/21/prepub
